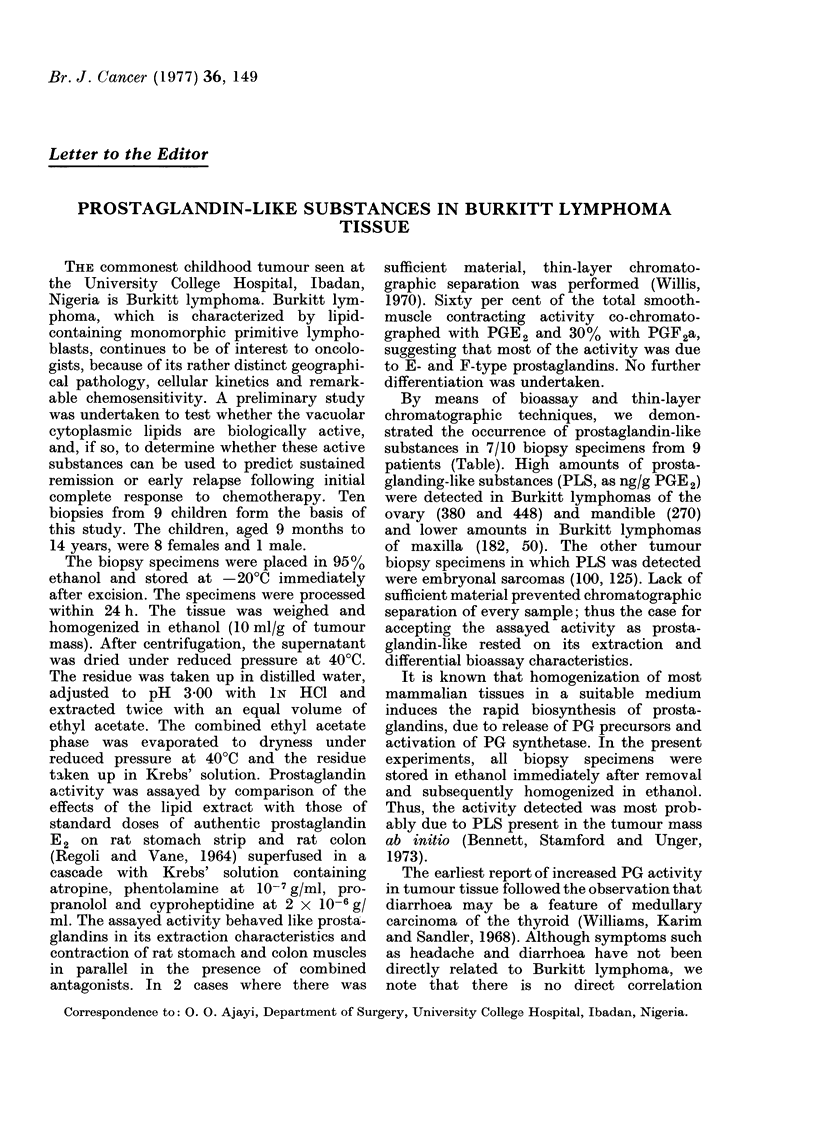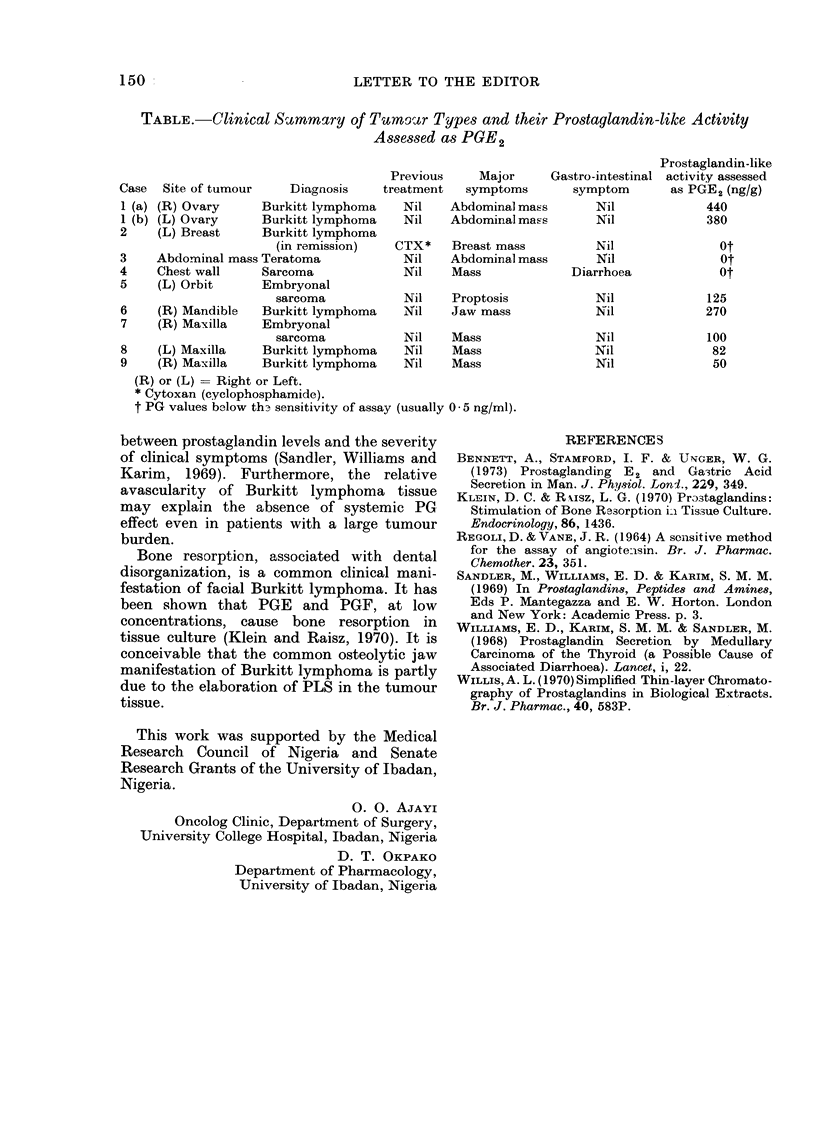# Prostaglandin-like substances in Burkitt lymphoma tissue.

**DOI:** 10.1038/bjc.1977.165

**Published:** 1977-07

**Authors:** O. O. Ajayi, D. T. Okpako


*Br. J. Cancer* (1977) **36**, 149

### Letter to the Editor

### PROSTAGLANDIN-LIKE SUBSTANCES IN BURKITT LYMPHOMA TISSUE

THE commonest childhood tumour seen at the University College Hospital, Ibadan, Nigeria is Burkitt lymphoma. Burkitt lymphoma, which is characterized by lipid-containing monomorphic primitive lymphoblasts, continues to be of interest to oncologists, because of its rather distinct geographical pathology, cellular kinetics and remarkable chemosensitivity. A preliminary study was undertaken to test whether the vacuolar cytoplasmic lipids are biologically active, and, if so, to determine whether these active substances can be used to predict sustained remission or early relapse following initial complete response to chemotherapy. Ten biopsies from 9 children form the basis of this study. The children, aged 9 months to 14 years, were 8 females and 1 male.

The biopsy specimens were placed in 95% ethanol and stored at −20°C immediately after excision. The specimens were processed within 24 h. The tissue was weighed and homogenized in ethanol (10 ml/g of tumour mass). After centrifugation, the supernatant was dried under reduced pressure at 40°C. The residue was taken up in distilled water, adjusted to pH 3·00 with 1N HCl and extracted twice with an equal volume of ethyl acetate. The combined ethyl acetate phase was evaporated to dryness under reduced pressure at 40°C and the residue taken up in Krebs' solution. Prostaglandin activity was assayed by comparison of the effects of the lipid extract with those of standard doses of authentic prostaglandin $E_2$ on rat stomach strip and rat colon (Regoli and Vane, 1964) superfused in a cascade with Krebs' solution containing atropine, phentolamine at $10^{-7}$ g/ml, propranolol and cyproheptidine at $2 \times 10^{-6}$ g/ml. The assayed activity behaved like prostaglandins in its extraction characteristics and contraction of rat stomach and colon muscles in parallel in the presence of combined antagonists. In 2 cases where there was sufficient material, thin-layer chromatographic separation was performed (Willis, 1970). Sixty per cent of the total smooth-muscle contracting activity co-chromatographed with $PGE_2$ and 30% with $PGF_2a$, suggesting that most of the activity was due to E- and F-type prostaglandins. No further differentiation was undertaken.

By means of bioassay and thin-layer chromatographic techniques, we demonstrated the occurrence of prostaglandin-like substances in 7/10 biopsy specimens from 9 patients (Table). High amounts of prostaglanding-like substances (PLS, as ng/g $PGE_2$) were detected in Burkitt lymphomas of the ovary (380 and 448) and mandible (270) and lower amounts in Burkitt lymphomas of maxilla (182, 50). The other tumour biopsy specimens in which PLS was detected were embryonal sarcomas (100, 125). Lack of sufficient material prevented chromatographic separation of every sample; thus the case for accepting the assayed activity as prostaglandin-like rested on its extraction and differential bioassay characteristics.

It is known that homogenization of most mammalian tissues in a suitable medium induces the rapid biosynthesis of prostaglandins, due to release of PG precursors and activation of PG synthetase. In the present experiments, all biopsy specimens were stored in ethanol immediately after removal and subsequently homogenized in ethanol. Thus, the activity detected was most probably due to PLS present in the tumour mass *ab initio* (Bennett, Stamford and Unger, 1973).

The earliest report of increased PG activity in tumour tissue followed the observation that diarrhoea may be a feature of medullary carcinoma of the thyroid (Williams, Karim and Sandler, 1968). Although symptoms such as headache and diarrhoea have not been directly related to Burkitt lymphoma, we note that there is no direct correlation

Correspondence to: O. O. Ajayi, Department of Surgery, University College Hospital, Ibadan, Nigeria.

TABLE.—*Clinical Summary of Tumour Types and their Prostaglandin-like Activity Assessed as $PGE_2$*

| Case | Site of tumour | Diagnosis | Previous treatment | Major symptoms | Gastro-intestinal symptom | Prostaglandin-like activity assessed as $PGE_2$ (ng/g) |
|---|---|---|---|---|---|---|
| 1 (a) | (R) Ovary | Burkitt lymphoma | Nil | Abdominal mass | Nil | 440 |
| 1 (b) | (L) Ovary | Burkitt lymphoma | Nil | Abdominal mass | Nil | 380 |
| 2 | (L) Breast | Burkitt lymphoma (in remission) | CTX* | Breast mass | Nil | 0† |
| 3 | Abdominal mass | Teratoma | Nil | Abdominal mass | Nil | 0† |
| 4 | Chest wall | Sarcoma | Nil | Mass | Diarrhoea | 0† |
| 5 | (L) Orbit | Embryonal sarcoma | Nil | Proptosis | Nil | 125 |
| 6 | (R) Mandible | Burkitt lymphoma | Nil | Jaw mass | Nil | 270 |
| 7 | (R) Maxilla | Embryonal sarcoma | Nil | Mass | Nil | 100 |
| 8 | (L) Maxilla | Burkitt lymphoma | Nil | Mass | Nil | 82 |
| 9 | (R) Maxilla | Burkitt lymphoma | Nil | Mass | Nil | 50 |

(R) or (L) = Right or Left.
* Cytoxan (cyclophosphamide).
† PG values below the sensitivity of assay (usually 0·5 ng/ml).

between prostaglandin levels and the severity of clinical symptoms (Sandler, Williams and Karim, 1969). Furthermore, the relative avascularity of Burkitt lymphoma tissue may explain the absence of systemic PG effect even in patients with a large tumour burden.

Bone resorption, associated with dental disorganization, is a common clinical manifestation of facial Burkitt lymphoma. It has been shown that PGE and PGF, at low concentrations, cause bone resorption in tissue culture (Klein and Raisz, 1970). It is conceivable that the common osteolytic jaw manifestation of Burkitt lymphoma is partly due to the elaboration of PLS in the tumour tissue.

This work was supported by the Medical Research Council of Nigeria and Senate Research Grants of the University of Ibadan, Nigeria.

O. O. AJAYI
Oncolog Clinic, Department of Surgery,
University College Hospital, Ibadan, Nigeria
D. T. OKPAKO
Department of Pharmacology,
University of Ibadan, Nigeria

REFERENCES

BENNETT, A., STAMFORD, I. F. & UNGER, W. G. (1973) Prostaglanding $E_2$ and Gastric Acid Secretion in Man. *J. Physiol. Lond.*, **229**, 349.

KLEIN, D. C. & RAISZ, L. G. (1970) Prostaglandins: Stimulation of Bone Resorption in Tissue Culture. *Endocrinology*, **86**, 1436.

REGOLI, D. & VANE, J. R. (1964) A sensitive method for the assay of angiotensin. *Br. J. Pharmac. Chemother.* **23**, 351.

SANDLER, M., WILLIAMS, E. D. & KARIM, S. M. M. (1969) In *Prostaglandins, Peptides and Amines*, Eds P. Mantegazza and E. W. Horton. London and New York: Academic Press. p. 3.

WILLIAMS, E. D., KARIM, S. M. M. & SANDLER, M. (1968) Prostaglandin Secretion by Medullary Carcinoma of the Thyroid (a Possible Cause of Associated Diarrhoea). *Lancet*, i, 22.

WILLIS, A. L. (1970) Simplified Thin-layer Chromatography of Prostaglandins in Biological Extracts. *Br. J. Pharmac.*, **40**, 583P.